# Assessment of Antimicrobial Activity and Safety of *Pediococcus pentosaceus* Isolated from Ginseng as a Functional Cosmetic Ingredient

**DOI:** 10.3390/microorganisms13051093

**Published:** 2025-05-08

**Authors:** Xiangji Jin, Qiwen Zheng, Trang Thi Minh Nguyen, Gyeong-Seon Yi, Su-Jin Yang, Tae-Hoo Yi

**Affiliations:** 1 Department of Dermatology, Graduate School, Kyung Hee University, 26 Kyungheedae-ro, Dong-daemun, Seoul 02447, Republic of Korea; hyanghe112@khu.ac.kr; 2 Graduate School of Biotechnology, Kyung Hee University, 1732 Deogyeong-daero, Giheung-gu, Yongin-si 17104, Republic of Korea; zhengqiwen@khu.ac.kr (Q.Z.); trangnguyen@khu.ac.kr (T.T.M.N.); stella@khu.ac.kr (S.-J.Y.); 3 Department of Biopharmaceutical Biotechnology, Graduate School, Kyung Hee University, 1732 Deogyeong-daero, Giheung-gu, Yongin-si 17104, Republic of Korea; ks010924@khu.ac.kr

**Keywords:** lactic acid bacteria, antimicrobial activity, safety, *G. mellonella* larvae, *Pediococcus pentosaceus*

## Abstract

Lactic acid bacteria (LAB) are gaining increasing attention as functional ingredients in the cosmetic industry, particularly those derived from natural plant sources. Although various LAB strains have been widely applied in cosmetic formulations, studies investigating the effects of naturally derived LAB on the skin remain limited. In this study, we isolated an LAB strain from ginseng and evaluated its potential as a functional cosmetic ingredient. The antimicrobial activity of the strain was assessed against skin-associated pathogens *Staphylococcus aureus* and *Staphylococcus epidermidis*, while cytotoxicity was evaluated using HaCaT and Caco-2 cells. Considering the limitations of vertebrate animal testing, infection and survival assays were conducted using *Galleria mellonella* larvae as an alternative in vivo model. The ginseng-derived strain exhibited 99.93% similarity to *Pediococcus pentosaceus* and was designated *P. pentosaceus* THG-219. It exhibited an MIC of 0.625 mg/mL and 1.25 mg/mL against *S. aureus* KCTC 3881 and *S. epidermidis* KCTC 1917, respectively. Its antimicrobial activity was further enhanced following ethyl acetate fractionation. *P. pentosaceus* THG-219 showed no toxicity in *G. mellonella* larvae and exerted antibacterial effects in this model. No cytotoxicity was observed in HaCaT and Caco-2 cells. Furthermore, *P. pentosaceus* THG-219 promoted host cell adhesion while inhibiting pathogen adhesion. It also exhibited excellent acid, bile, and heat tolerance, suggesting strong survivability under harsh conditions. Collectively, these results indicate that *P. pentosaceus* THG-219, isolated from ginseng, is a promising, safe, and stable candidate for development as a functional cosmetic ingredient.

## 1. Introduction

The human skin microbiome comprises a diverse community of microorganisms, including commensals and opportunistic pathogens [1]. Among them, *Staphylococcus epidermidis* and *Staphylococcus aureus* are the most extensively studied bacterial species due to their dual roles in maintaining skin health and contributing to disease. *S. aureus* is a well-known opportunistic pathogen frequently associated with various skin disorders such as atopic dermatitis, folliculitis, impetigo, and wound infections. It produces a wide array of virulence factors, including exotoxins, proteases, and biofilm-forming capabilities, which disrupt the skin barrier, induce inflammation, and contribute to chronic skin conditions [2]. In individuals with compromised skin integrity or dysbiosis of the cutaneous microbiota, *S. aureus* can proliferate excessively, exacerbating symptoms and increasing susceptibility to secondary infections. On the other hand, *S. epidermidis* is traditionally regarded as a commensal and beneficial component of the skin microbiota but is also known to exhibit complex behaviors that can contribute to either skin protection or pathology depending on the context [3].

Therefore, a critical goal in dermatological and cosmetic science is to selectively suppress pathogenic or overabundant strains of *S. aureus* and *S. epidermidis* without disturbing the beneficial balance of the skin microbiome [4]. With the growing demand for natural and microbiome-friendly cosmetic products, LAB have garnered significant attention as potential active ingredients in formulations aimed at managing acne, atopic dermatitis, photoaging, and sensitive skin [5]. Recent studies have demonstrated that probiotics such as *Lactobacillus* and *Lactiplantibacillus* exhibit a variety of skin-improving effects, including skin moisturization, anti-aging, anti-inflammatory, and whitening effects, making them increasingly recognized for their potential as cosmetic ingredients [6]. For instance, *Lactobacillus rhamnosus* GG has been reported to modulate skin inflammation and improve barrier function by reducing transepidermal water loss and enhancing tight junction formation in keratinocytes [7]. *Lactobacillus plantarum* has demonstrated notable antioxidant and wound-healing effects in both in vitro and in vivo models, supporting its use in anti-aging and skin regeneration applications [8]. In addition, strains such as *Lactobacillus reuteri* [9] and *Streptococcus thermophilus* [10] have been commercialized in various topical formulations due to their ability to enhance ceramide synthesis and improve skin hydration. These examples reflect the increasing interest in exploiting the dermatological potential of LAB beyond their traditional probiotic roles in gut health and highlight the importance of identifying novel, safe, and functional strains for cosmetic use [11].

Despite their commercial introduction, many of these probiotic strains still face limitations due to inconsistent growth, suboptimal antimicrobial efficacy, and safety concerns associated with the use of live microorganisms [12]. Moreover, ethical considerations have led regulatory authorities, including those in the European Union, to impose strict bans on the use of vertebrate animals in cosmetic testing [13]. This shift has accelerated the global development and adoption of alternative testing models. Currently, most LAB strains used in research and product development are derived from fermented foods or dairy products, while those isolated from medicinal plants or natural habitats remain underexplored [14]. Such strains may offer unique bioactive properties and better compatibility with skin applications—especially when derived from traditional herbal resources like ginseng, which has well-documented pharmacological effects [15]. The search for safe and effective antimicrobial agents, particularly those originating from probiotics or plant-based sources, holds promise for microbiome-compatible cosmetics that promote skin health, prevent infection, and reduce inflammation [16].

In this context, we aimed to isolate LAB from the medicinal plant ginseng and screen for strains with beneficial dermatological properties. Our goal was to identify a safe, stable, and effective candidate capable of inhibiting skin pathogens while being non-toxic and potentially beneficial when applied topically or ingested. Ultimately, we sought to demonstrate its potential as a functional ingredient in skin-targeted cosmetic formulations.

## 2. Materials and Methods

### 2.1. Isolation and Identification of LAB from Ginseng

The ginseng extract was serially diluted in 0.85% (*w*/*v*) saline to concentrations ranging from 10^−6^ to 10^−9^ and then spread onto bromocresol purple (BCP; Eiken Chemical Co., Ltd., Tokyo, Japan) agar plates. The plates were incubated at 37 °C for 2 days. Single colonies surrounded by yellow halos on the BCP agar were selected and subcultured onto De Man, Rogosa, and Sharpe (MRS) agar (Difco, Detroit, MI, USA) plates, followed by incubation in both MRS agar and MRS broth at 30 °C.

To identify the isolated strains, 16S rRNA gene sequencing was performed using the primers 1492R (5′-CGAAACCGCACAGTGGTTTT-3′) and 27F (5′-GGGGATACGGGGTGCTATACAT-3′). The obtained 16S rRNA sequences were analyzed using the EzBioCloud database (https://www.ezbiocloud.net/identify, accessed on 6 March 2024). Phylogenetic trees were constructed using the neighbor-joining and maximum-parsimony methods in the MEGA X version 7.0 (Molecular Evolutionary Genetics Analysis), and bootstrap values were calculated based on 1000 replications [17]. For biochemical analysis, carbohydrate fermentation tests were conducted using the API 50 CHL kit (BioMérieux, Marcy l’Etoile, France) [18].

### 2.2. Bacterial Strains and Culture Conditions

*Staphylococcus aureus* KCTC 3881, *Staphylococcus epidermidis* KCTC 1917, *Lacticaseibacillus rhamnosus* KCTC 5032, and *Pediococcus pentosaceus* KCTC 12311 were obtained from the Korean Collection for Type Cultures (KCTC). The strains were cultured on nutrient broth (NB; Difco, Detroit, MI, USA) agar plates under aerobic conditions at 30 °C. LAB were cultured in MRS medium at 30 °C for 24 h. Following incubation, the cultures were centrifuged at 3500 rpm for 10 min at 4 °C, and the resulting supernatant was filtered through a 0.22 μm membrane filter to obtain the cell-free supernatant (CFS). For extraction, the CFS was mixed with an equal volume of ethyl acetate (1:1, *v*/*v*) and allowed to stand for 24 h. This extraction step was repeated twice. The ethyl acetate layer was collected and concentrated using a rotary vacuum evaporator at 40 °C under reduced pressure of approximately 100 mbar for 30 min (EYELA WORLD—Tokyo Rikakikai Co., Ltd., Tokyo, Japan), yielding the ethyl acetate fraction (EA), which was recovered as a concentrated extract (1.75% *w*/*w*) and stored at 4 °C until use. The remaining aqueous layer, which was not soluble in ethyl acetate, was defined as the aqueous fraction (AQ). This fraction was filtered through a qualitative filter paper (Whatman™, Cytiva, Tokyo, Japan), concentrated using the same rotary vacuum evaporator, and stored as a concentrated extract (2.15% *w*/*w*) at 4 °C.

### 2.3. Disk Diffusion Assay

LAB with antimicrobial properties were evaluated using the standard disk diffusion method according to the protocol by Zaidan et al. [19]. Briefly, 100 µL of the filtered supernatant was applied to sterile filter paper disks (8 mm in diameter, Whatman No. 1), which were then placed onto NB agar plates previously inoculated with indicator pathogens (1 × 10^6^ CFU/mL). The plates were incubated at 30 °C for 24 h. The antimicrobial activity of the LAB was assessed by measuring the diameter of the inhibition zones in millimeters using a caliper [20].

### 2.4. Broth Microdilution Assay

The minimum inhibitory concentration (MIC) and minimum bactericidal concentration (MBC) of the selected LAB were determined using the broth microdilution method, as described in the guidelines of the Clinical and Laboratory Standards Institute (CLSI) [21]. Serially diluted evaporated CFSs of the selected LAB strains were inoculated into a 96-well microtiter plate (Thermo Fisher Scientific, Waltham, MA, USA). Each well contained 100 μL of the serially diluted CFS and 100 μL of a suspension of *S. aureus* KCTC 3881 or *S. epidermidis* KCTC 1917 (1 × 10^6^ CFU/mL) in NB. The plates were incubated at 37 °C for 24 h. After incubation, the optical density (OD) at 595 nm was measured using a microplate reader (Molecular Devices, San Francisco, CA, USA). To determine the MBC, 1 μL from each well was streaked onto NB agar plates using a sterile inoculation loop. The plates were incubated at 37 °C for 24 h, and the lowest concentration at which no visible bacterial colonies were observed was recorded as the MBC.

### 2.5. Biofilm Formation Inhibition Assay

To assess the anti-biofilm activity of evaporated CFS from THG-219, 100 μL of diluted pathogenic bacteria (1 × 10^6^ CFU/mL) was mixed with an equal volume of CFS at various concentrations (0–5 mg/mL) in a 96-well plate. After 24 h incubation at 37 °C without shaking, wells were gently washed twice with PBS to remove planktonic cells. Biofilms were stained with 0.01% crystal violet for 15 min, washed again, and air-dried. The bound dye was solubilized using 33% acetic acid, and OD at 595 nm was measured. Evaporated MRS broth served as the control [22].

### 2.6. Scanning Electron Microscopy (SEM) of Bacteria

Diluted pathogen suspensions at a concentration of 1 × 10^7^ CFU/mL were treated with 2× the MIC of the evaporated CFS at 37 °C for 24 h. The pathogens were then fixed in PBS containing 2.5% glutaraldehyde at 4 °C for 3 h. The fixed samples were dehydrated using a graded ethanol series (30%, 50%, 70%, 80%, 90%, and 100%). After dehydration, 100 μL of hexamethyldisilazane (Sigma Aldrich, St. Louis, MO, USA) was added, and the bacteria were dried overnight. A carbon tape was attached to the SEM stub, and the dried samples were mounted on it. A plasma coating was applied to generate a metallic film on the surface of the non-conductive samples. The bacteria were visualized using a SU8010 scanning electron microscope (Hitachi, Tokyo, Japan).

### 2.7. Cell Culture and Viability

HaCaT and Caco-2 cells were obtained from the Korean Cell Line Bank (KCLB) and cultured in DMEM supplemented with 10% FBS and 1% penicillin at 37 °C in a 5% CO_2_ incubator. To assess cell viability following treatment with THG-219 CFS and EA, MTT assay was performed on HaCaT cells, and LDH assay was conducted on Caco-2 cells. Absorbance was measured at 595 nm using a FilterMax F5 microplate reader (Molecular Devices) [23].

### 2.8. Adhesion Assay and Anti-Adhesion Activity of THG-219

To assess the cell adhesion activity of THG-219, Caco-2 and HaCaT cells were seeded in 24-well plates and cultured until confluence was reached (80–90% confluence). Afterward, THG-219 and *Lactobacillus rhamnosus* KCTC 5032 were added to the cells, and the cells were incubated for 2 h. To remove non-adherent bacteria, the wells were washed with PBS, and the cells were fixed, stained with crystal violet, and treated with 33% acetic acid. Adhesion was quantified by measuring absorbance at 595 nm [24].

For the inhibition assay, THG-219 CFS was co-incubated with Caco-2 and HaCaT cells, which had grown to confluence in 24-well plates, for 1 h. Following this, *S. aureus* or *S. epidermidis* suspensions (1 × 10^8^ CFU/mL) were added, and the cells were incubated at 37 °C for an additional 2 h. Non-adherent bacteria were removed by washing with PBS, and the cells were fixed, stained with crystal violet, and treated with 33% acetic acid. Adhesion was quantified by measuring absorbance at 595 nm.

### 2.9. Galleria mellonella Larval Model for Toxicity and Antimicrobial Activity

The in vivo toxicity and antimicrobial efficacy of THG-219 against *S. aureus* and *S. epidermidis* were evaluated using the *Galleria mellonella* larval model. Larvae (200–240 mg) were injected with 10 μL of *S. aureus* or *S. epidermidis* (10^6^ CFU/mL) into the left proleg [25]. After 6 h, 10 μL of CFS or EA of THG-219 (0.006 mg/mL) was injected. PBS (10 μL) was used as a negative control. Four larvae were used per condition, and experiments were repeated three times. Survival was monitored over 96 h at 37 °C, and death was defined as the absence of movement in response to gentle stimulation.

### 2.10. Acid, Bile, and Heat Tolerance Assay

To assess acid resistance, an artificial gastric environment was simulated by adjusting MRS broth to pH 2.5. The strain THG-219 was then introduced at a concentration of 1 × 10^6^ CFU/mL. At specified time intervals (0, 15, 30, 60, 120, and 180 min), samples were taken and subjected to serial 10-fold dilutions using 0.85% NaCl. Aliquots from dilutions ranging from 10^−4^ to 10^−8^ were plated in triplicate onto MRS agar. After incubation at 37 °C for 24 h, the number of viable colonies was counted, and bacterial survival was analyzed over time in comparison to the initial population [26].

Bile resistance was examined by exposing the strain THG-219 to MRS broth supplemented with 0.3% (*w*/*v*) bile salts (Sigma, St. Louis, MO, USA), simulating intestinal conditions. The bacterial culture, adjusted to 1 × 10^6^ CFU/mL, was incubated, and samples were collected at 0, 15, 30, 60, 120, and 180 min. Each sample was serially diluted in 0.85% NaCl following a 10-fold dilution protocol. From each dilution (10^−4^ to 10^−8^), aliquots were spread in triplicate onto MRS agar plates. Following a 24 h incubation at 37 °C, the number of colony-forming units (CFUs) was counted to determine the survival rate over time relative to the initial bacterial load [27].

To determine thermal stability, THG-219 cultures suspended in phosphate-buffered saline (PBS) were subjected to heat exposure at incremental temperatures (30 °C, 40 °C, 50 °C, 60 °C, 70 °C, and 80 °C) for a duration of 30 min each. Following the thermal challenge, the samples underwent serial dilution and were plated onto MRS agar. The inoculated plates were incubated at 37 °C for 48 h, after which colony counts were performed to evaluate bacterial survival at each temperature [28].

### 2.11. Statistical Analysis

All statistical evaluations were carried out using GraphPad Prism version 9.0 (GraphPad Software, La Jolla, CA, USA). Experimental results from three biologically independent replicates are expressed as means ± standard deviation (SD). Differences between groups were analyzed using one-way or two-way analysis of variance (ANOVA), where appropriate. A *p*-value of less than 0.05 was considered statistically significant, with levels of significance denoted as *p* < 0.05, *p* < 0.01, and *p* < 0.001.

## 3. Results

### 3.1. Identification and Biological Characteristics of Strain THG-219

Phylogenetic analysis based on 16S rRNA gene sequencing revealed that strain THG-219 shares the highest sequence similarity (99.93%) with its closest relative, *P. pentosaceus* DSM 20336^T^. Based on this result, comparative biochemical and cultural characteristics were assessed using *P. pentosaceus* DSM 20336^T^, which also exhibited high sequence similarity. In the API 50CHL test, all strains were negative for erythritol, D-arabinose, L-arabinose, D-ribose, D-xylose, L-xylose, D-adonitol, methyl-β-D-xylopyranoside, L-sorbose, L-rhamnose, dulcitol, inositol, D-mannitol, D-sorbitol, methyl-αD-mannopyranoside, methyl-αD-glucopyranoside, D-lactose, D-melibiose, D-saccharose, inulin, D-melezitose, D-raffinose, starch, glycogen, xylitol, D-turanose, D-lyxose, D-fucose, L-fucose, D-arabitol, L-arabitol, potassium gluconate, potassium 2-ketogluconate, and potassium 5-ketogluconate. Notably, *P. pentosaceus* THG-219 differs from the reference strain DSM 20336^T^ by its ability to ferment glycerol, D-xylose, and D-raffinose, while lacking the ability to ferment lactose, melibiose, and sucrose, indicating a distinct carbohydrate utilization pattern (Table 1).

**Figure 1:**
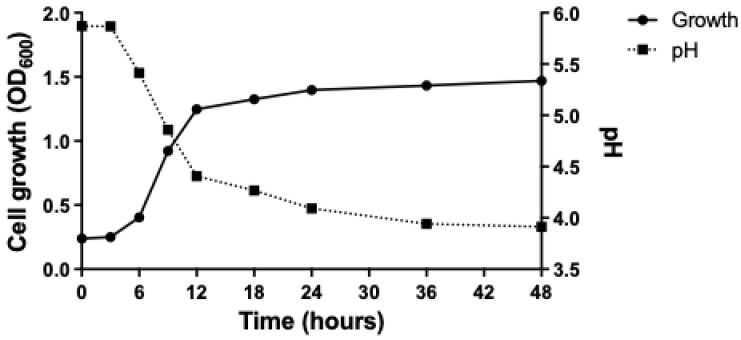
Growth curve and pH effect of *P. pentosaceus* THG-219.

*P. pentosaceus* THG-219 exhibited unique growth dynamics during the cultivation process. After an initial period, the strain experienced a lag phase of approximately 6 h, indicating an adaptation phase characterized by limited proliferation. Subsequently, the strain entered an active exponential growth phase between 6 and 24 h post-inoculation, marked by a substantial increase in cell population under favorable conditions. At 48 h post-inoculation, the pH of the culture medium stabilized at 3.9 pH, serving as an important indicator of the strain’s metabolic activity and the prevailing environmental conditions within the medium. These findings provide valuable insights into the growth kinetics and pH homeostasis mechanisms of *P. pentosaceus* THG-219 and define its optimal growth conditions (Figure 1).

### 3.2. Antibacterial Activity of THG-219

*P. pentosaceus* THG-219 exhibited significant antibacterial activity against *S. epidermidis* KCTC 1917 and *S. aureus* KCTC 3881. The MIC values were determined to be 1.25 mg/mL and 0.625 mg/mL, respectively. Notably, the MIC value of THG-219 against *S. epidermidis* was lower than that of the reference strain *P. pentosaceus* KACC 12311, which exhibited an MIC of 2.5 mg/mL against the same pathogen. Both THG-219 and the reference strain showed MBC values of 5 mg/mL. These results indicate that THG-219 possesses enhanced antibacterial activity, particularly against *S. epidermidis*, as presented in Table 2.

### 3.3. Inhibition of Biofilm Formation by THG-219

**Figure 2:**
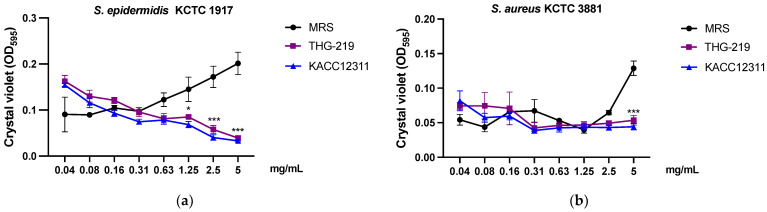
Anti-biofilm-formation activities of strain against *P. pentosaceus* THG-219 and KACC 12311 associated with (**a**) *S. epidermidis* KCTC 1917, (**b**) *S. aureus* KCTC 3881. Data are presented as mean ± SD of the result in three replicates. *, *p* < 0.05, ***, *p* < 0.001 compared to control.

As shown in Figure 2, the CFS of *P. pentosaceus* THG-219 and *P. pentosaceus* KACC 12311 effectively inhibited biofilm formation by *S. aureus* KCTC 39881 and *S. epidermidis* KCTC 1917. To further investigate this inhibitory activity, a dose–response analysis was performed. Both supernatants suppressed biofilm formation by *S. aureus* KCTC 3881 in a concentration-dependent manner; however, the low R^2^ values (TH-G219: y = −0.0028x + 0.0607, R^2^ = 0.1263; KACC 12311: y = −0.0035x + 0.0557, R^2^ = 0.1772) indicate a weak correlation between concentration and inhibition. Similarly, a dose-dependent inhibitory effect on *S. epidermidis* KCTC 1917 was observed (TH-G219: y = −0.0175x + 0.104, R^2^ = 0.5867), whereas the MRS control exhibited a strong positive correlation with enhanced biofilm formation (y = 0.0229x + 0.0994, R^2^ = 0.9109). Although both strains showed moderate anti-biofilm activity, the lack of a strong concentration–effect relationship and the absence of statistically significant differences between the two suggest that they may exert similar effects through shared physiological and biochemical mechanisms, as both strains belong to the same species.

### 3.4. SEM Observation of Morphological Alterations in Bacterial Cells

**Figure 3:**
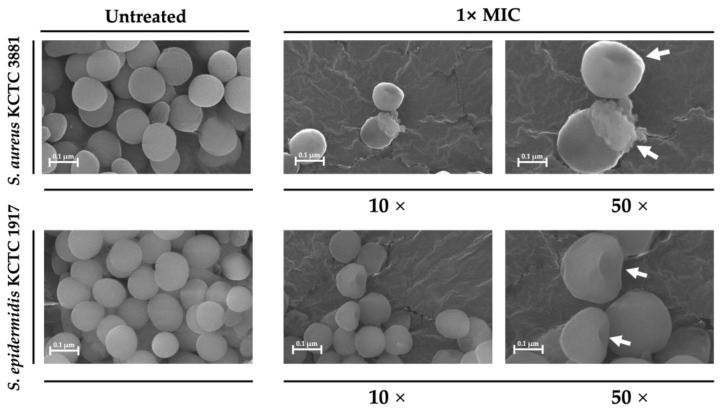
Scanning electron microscopy images (magnification: 100,000×, 500,000×; scale bar: 0.1 µm) confirming the antimicrobial effects of the THG-219 CFS *on S. epidermidis* KCTC 1917, *S. aureus* KCTC 3881. The white arrows illustrate the extent and manner of bacterial cell death.

As shown in Figure 3, morphological changes on the cell surface induced by THG-219 at 1× MIC were examined using SEM. Under untreated conditions, the pathogenic bacteria exhibited uniform, spherical shapes with intact cell walls. However, after 24 h of exposure to the CFS of THG-219, both *S. epidermidis* KCTC 1917 and *S. aureus* KCTC 3881 showed severe structural damage, including deformation of the cell membrane, resulting in a marked alteration from the typical *Staphylococcus* morphology. These observations suggest that THG-219 causes damage to the cell wall and membrane, compromising the structural integrity of the pathogens.

### 3.5. Antibacterial Activity of Purified THG-219 Against Pathogens Assessed by Disk Diffusion

**Figure 4:**
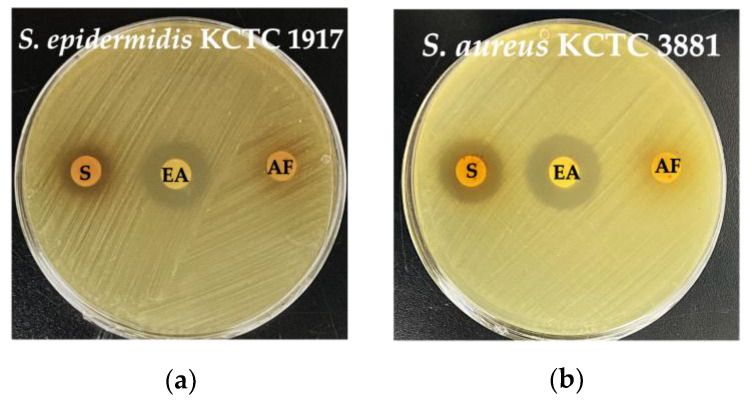
Antibacterial activity of THG-219 CFS and its EA fractions by disk diffusion assay. (**a**) Inhibition zone bar graph of CFS and EA against *S. epidermidis* KCTC 1917; (**b**) Inhibition zone bar graph of CFS and EA against *S. aureus* KCTC 3881. S: THG-219 CFS; EA: EA fraction of the THG-219 supernatant; AF: aqueous fraction (insoluble in ethyl acetate).

The antibacterial activity of THG-219 against *S. epidermidis* KCTC 1917 and *S. aureus* KCTC 3881 was confirmed through a broth microdilution assay. To purify the active compound, an EA fractionation was performed, and its antibacterial efficacy was evaluated in comparison with the crude CFS (Figure 4, Table 3). In the disk diffusion assay, the application of 100 µL of the EA fraction at a concentration of 20 mg/mL resulted in inhibition zones of 20 mm for *S. epidermidis* KCTC 1917 and 22 mm for *S. aureus* KCTC 3881. In contrast, the inhibition zones observed with 100 µL of the crude CFS were 15 mm for *S. epidermidis* KCTC 1917 and 19 mm for *S. aureus* KCTC 3881.

### 3.6. Cytotoxic Effect of THG-29

**Figure 5:**
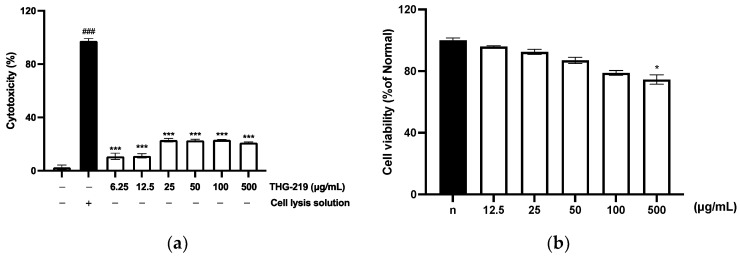
Cytotoxic effects of THG-219 CFS. (**a**) Cytotoxicity assessment of THG-219 on Caco-2 cells using the LDH assay; (**b**) Dose-dependent cytotoxicity of THG-219 on HaCaT cells using the MTT assay. Data are presented as mean ± SD of the result in three replicates. ### *p* < 0.001 compared to the un-treated group. *, *p* < 0.05, ***, *p* < 0.001 compared to the control group.

As shown in Figure 5, cytotoxicity tests of THG-219 CFS were conducted on Caco-2 cells using the Dyne LDH PLUS Cytotoxicity Assay Kit (Dynebio Inc., Seoul, Republic of Korea) and on HaCaT cells using the MTT assay (Sigma-Aldrich, St. Louis, MO, USA). When treated with 500 μg/mL, no cytotoxicity was observed in Caco-2 cells, as shown in Figure 5a. However, in HaCaT cells (Figure 5b), cytotoxic effects were evident at the highest concentration (500 μg/mL), while no significant cytotoxicity was detected at concentrations up to 100 μg/mL. These findings suggest that 100 μg/mL may serve as a suitable maximum concentration for future in vitro studies.

### 3.7. Adhesion Ability and Anti-Adhesion Ability of THG-219 to Caco-2 and HaCaT Cells

**Figure 6:**
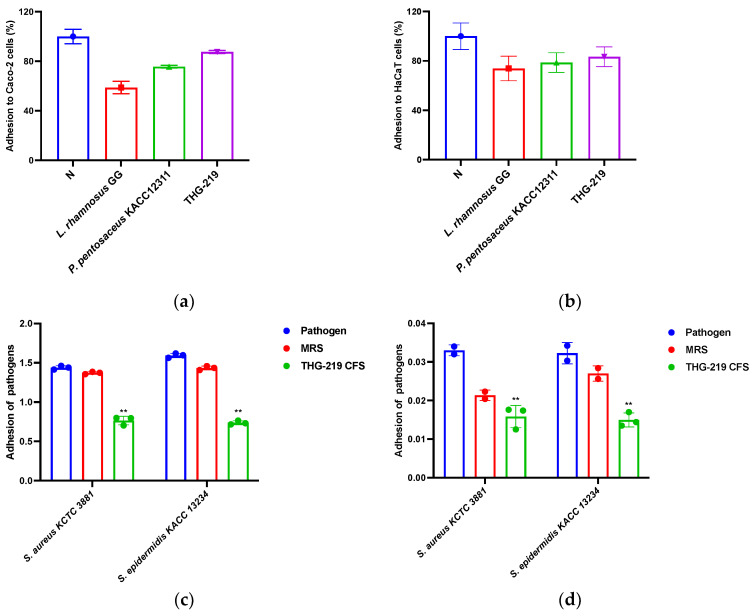
Cell adhesion ability of THG-219 and its inhibitory effects on pathogen adhesion. (**a**) Adhesion of THG-219 to Caco-2 cells; (**b**) Adhesion of THG-219 to HaCaT cells; (**c**) Inhibitory effects of THG-219 on the adhesion of *S. epidermidis* KCTC 1917 and *S. aureus* KCTC 3881 to Caco-2 cells; (**d**) Inhibitory effects of THG-219 on the adhesion of *S. epidermidis* KCTC 1917 and *S. aureus* KCTC 3881 to HaCaT cells. Data are presented as mean ± SD of the result in three replicates. **, *p* < 0.01 compared to the control group.

As shown in Figure 6a,b, THG-219 showed significantly higher adhesion to Caco-2 cells than the control strain *L. rhamnosus* GG KCTC 5032. In particular, the adhesion ability of THG-219 was superior to that of its isogenic strain *P. pentosaceus* KACC 12311. Similar patterns were observed in HaCaT cells, where THG-219 consistently showed improved adhesion. These results suggest that the strong adhesion ability of THG-219 may contribute to the improved stability and efficacy in host-related applications.

In addition, THG-219 effectively inhibited the adhesion of pathogenic bacteria *S. epidermidis* KCTC 1917 and *S. aureus* KCTC 3881 to Caco-2 cells, showing an inhibition rate of 44.19%, 48.54% compared to the MRS control. A similar inhibitory effect was observed in HaCaT cells, with inhibition rates reaching 25.83%, 44.56% for both pathogens. These findings suggest the potential of THG-219 as an alternative to conventional antibiotics in preventing pathogenic bacterial colonization (Figure 6c,d).

### 3.8. Toxicity and Antimicrobial Evaluation of THG-219 Using the Galleria mellonella Larvae Infection Model

The survival of *G. mellonella* larvae treated with THG-219, as well as those infected with *S. aureus* and *S. epidermidis*, was evaluated. A 10 μL aliquot of bacterial culture or PBS (control) was injected into the second-to-last left proleg of each larva. Following injection, the larvae were incubated at 37 °C and monitored every 24 h for up to 7 days to assess survival. After a 12 h infection with *S. epidermidis* KCTC 1917 or *S. aureus* KCTC 3881, THG-219 was administered to the larvae, followed by incubation for up to 7 days.

**Figure 7:**
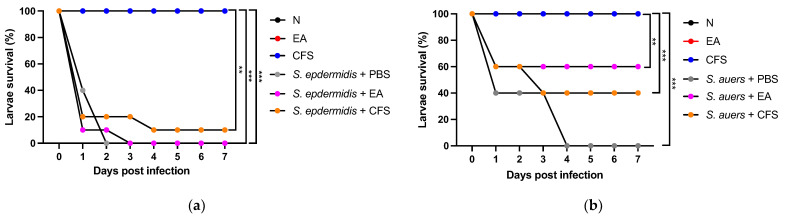
Survival assessment of *G. mellonella* larvae treated with THG-219. (**a**) Antibacterial effect of THG-219 on *G. mellonella* larvae infected with *S. epidermidis* KCTC 1917 and toxicity assessment of THG-219 in uninfected larvae, (**b**) Antibacterial effect of THG-219 on *G. mellonella* larvae infected with *S. aureus* KCTC 3881 and toxicity assessment of THG-219 in uninfected larvae. Data are presented as mean ± SD of the result in three replicates. **, *p* < 0.01, ***, *p* < 0.001 compared to the untreated group.

As shown in Figure 7, both the CFS and EA extract of THG-219 did not significantly reduce larval survival over the 7-day period, indicating no apparent toxicity. In contrast, larvae infected with *S. epidermidis,* or *S. aureus* alone exhibited complete mortality by day 2 or 4, respectively. Notably, larvae treated with the THG-219 EA extract after infection exhibited survival rates of 20% for *S. epidermidis* KCTC 1917 and 60% for *S. aureus* KCTC 3881, demonstrating its antibacterial efficacy. Of particular note, both THG-219 CFS and EA extract markedly improved the survival of larvae infected with *S. aureus*, underscoring the strong antimicrobial potential of THG-219 against this pathogen.

### 3.9. Acid, Bile, and Heat Tolerance of THG-219

**Figure 8:**
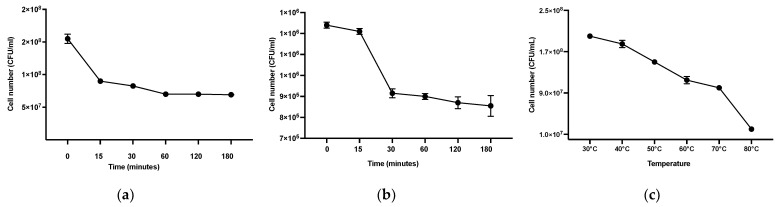
Assessment of acid tolerance, bile salt tolerance, and heat resistance of THG-219. (**a**) Viable cell counts of THG-219 under artificial acidic conditions (pH 2.5), (**b**) Viable cell counts of THG-219 under artificial bile salt conditions (0.3% bile salts), (**c**) Viable cell counts of THG-219 under high-temperature conditions (up to 80 °C). Data are presented as mean ± SD of the result in three replicates.

As shown in Figure 8, *P. pentosaceus* THG-219 exhibited approximately 54% survival after 120 min in artificial gastric juice at pH 2.5, indicating strong acid tolerance. This survival rate is significantly higher than that of *P. pentosaceus* M41, which survived for 2 h under acid stress at pH 3.0, as reported by Baig et al. [29].

Regarding bile tolerance, THG-219 maintained a survival rate of over 31% after 120 min of exposure to 0.3% bile salts. In contrast, *P. pentosaceus* MLK67 showed a survival level of over 10^5^ CFU/mL after incubation in 0.3% bile salts, as reported by Lim (Agricultural Information Resource Center) [30], while THG-219 demonstrated a higher viability of more than 8 × 10^5^ CFU/mL under the same conditions, confirming superior bile tolerance.

Furthermore, THG-219 exhibited a high survival rate of 50% even at 70 °C, suggesting substantial thermal tolerance. This is consistent with the findings of Raccach and Tilley, who reported that *P. pentosaceus* cultures experienced significant inactivation at temperatures above 65 °C [31].

## 4. Discussion

In this study, *P. pentosaceus* THG-219, isolated from ginseng, exhibited significant antimicrobial activity against skin-related pathogens *S. aureus* and *S. epidermidis*. The minimum inhibitory concentrations (MICs) were 0.625 mg/mL and 1.25 mg/mL, respectively, indicating that this strain is highly effective for topical applications. These results suggest that *P. pentosaceus* inhibits the growth of pH-sensitive pathogens such as *S. aureus* and *S. epidermidis* by producing lactic acid and acetic acid, which lower the pH of the culture medium. Additionally, *P. pentosaceus* has the ability to produce various bacteriocins (pediocin, penocin, sakacin, pentocin), which exhibit antimicrobial activity against specific bacterial strains. Notably, the antimicrobial activity was further enhanced after ethyl acetate fractionation (Figure 4). This enhancement is attributed to ethyl acetate’s ability to separate the aqueous phase containing water-soluble compounds from the organic solvent phase containing lipophilic compounds [32]. In this process, lipophilic compounds are separated and concentrated, while water-soluble compounds remain in the aqueous phase. Lipophilic antimicrobial compounds, such as polyphenols, lipid metabolites, certain bacteriocins, and fat-soluble vitamins, may contribute to the enhanced antimicrobial activity [33]. Furthermore, ethyl acetate aids in concentrating organic acids, which acidify the environment and further enhance the inhibitory effect on the growth of pathogenic microorganisms. These factors collectively explain the increased antimicrobial activity observed after fractionation.

Beyond antimicrobial properties, *P. pentosaceus* THG-219 demonstrated a dual-functional role by effectively inhibiting the attachment of pathogens while promoting adhesion to host cells. This dual effect is highly desirable in probiotic-based skin applications, as the ability to competitively exclude pathogens is essential for maintaining a balanced skin microbiota. Importantly, *P. pentosaceus* THG-219 adhered more strongly to HaCaT cells than *L. rhamnosus* GG, a well-established reference strain known for its high adhesion ability (Figure 6). This enhanced adhesion suggests superior colonization potential and prolonged skin residence, which may contribute to sustained protection. Furthermore, as shown in Figure 5, no cytotoxic effects were observed in either human-derived HaCaT keratinocytes or Caco-2 intestinal epithelial cells, indicating good biocompatibility with human tissues. These findings are consistent with previous reports demonstrating low toxicity of *Pediococcus* spp. in in vitro models [34].

Considering the ethical and regulatory restrictions associated with vertebrate testing in the cosmetics industry, the *G. mellonella* larval model presents a valuable alternative for in vivo toxicity and efficacy evaluations. This model enables simultaneous assessment of antimicrobial activity and safety within a living host while avoiding the complexities inherent in mammalian systems. In the present study, *P. pentosaceus* THG-219 exhibited potent antimicrobial activity against *S. epidermidis* KCTC 1917 and *S. aureus* KCTC 3881 without inducing toxicity in *G. mellonella* larvae (Figure 7). Although it may not fully replicate the reliability of animal or clinical studies, these findings support the utility of *G. mellonella* as a rapid, cost-effective, and ethically advantageous in vivo screening platform during the early stages of research. This model may serve as an important bridge toward subsequent validation in vertebrate models such as mice and potentially inform future clinical applications [35]. Previous studies have shown that the pathogenicity and survival rates observed in *G. mellonella* infected with pathogens like *S. aureus*, *P. aeruginosa*, and *A. baumannii* correlate well with outcomes in mammalian models [36]. Moreover, the ability of *G. mellonella* to survive at 37 °C enhances its relevance by allowing simulation of human infection conditions, including the expression of temperature-dependent virulence factors [37].

This strain also exhibited notable acid, bile, and heat tolerance (Figure 8), which are important indicators of probiotic and postbiotic functionality. As shown in Figure 8a, *P. pentosaceus* THG-219 survived for 3 h under simulated acidic conditions (pH 2.5) and maintained viability in artificial bile environments, despite more than 50% of cells dying after 30 min. This suggests that not all viable cells may reach the intestine under oral administration, and its survival in the human gastrointestinal tract remains uncertain. As shown in Figure 8c, the strain survived heat treatment up to 70 °C, with significant reduction occurring at 80 °C. Considering that cosmetic application typically occurs at ambient or skin temperatures around 37 °C, these results indicate that the strain or its postbiotic components are likely stable under relevant usage conditions [38].

The application of strains with broad antimicrobial activity to the skin may disrupt the delicate balance between beneficial and harmful microorganisms. Broad-spectrum antimicrobial agents can reduce beneficial bacteria such as *S. epidermidis* and *P. acnes*, which are essential for skin protection [39]. Such disruption can increase the risk of inflammation, infections, and chronic skin conditions. Long-term use of these strains may lead to decreased microbial diversity and impaired resilience of the skin microbiome [40]. However, certain probiotic strains, such as *Lactobacillus*, have been shown to restore microbial balance following disruption. Therefore, *P. pentosaceus* THG-219, which exhibits broad antimicrobial activity, may offer effective pathogen inhibition while also being safely applicable with consideration for skin microbiome balance. This strain may possess the ability to minimize its impact on beneficial skin microorganisms, making it a promising strategy for maintaining skin health. Thus, further studies are needed to closely evaluate the long-term effects of *P. pentosaceus* THG-219 on the skin microbiome.

Collectively, our findings support the potential of *P. pentosaceus* THG-219 as a multifunctional cosmetic ingredient with antimicrobial, adhesive, and stress-tolerant properties. However, several limitations remain. Although promising results were observed in vitro and in *G. mellonella*, further studies using advanced skin models or clinical trials are required to confirm efficacy and safety in humans. Additionally, the identification and characterization of specific bioactive compounds within the ethyl acetate fraction would help elucidate the molecular mechanisms underlying the observed effects. Finally, long-term stability and compatibility with other cosmetic ingredients should be assessed under real-world formulation and storage conditions. Addressing these limitations will strengthen the case for *P. pentosaceus* THG-219 as a novel, effective, and ethically viable ingredient for the future of cosmetic innovation.

## 5. Conclusions

In this study, *P. pentosaceus* THG-219 was isolated from ginseng and demonstrated its potential as a safe and effective functional ingredient for cosmetic applications. The strain exhibited notable antimicrobial activity against *S. aureus* and *S. epidermidis*, particularly after ethyl acetate fractionation. It showed no cytotoxicity in HaCaT and Caco-2 cells and was non-toxic in *G. mellonella* larvae, which also served as a useful in vivo model for evaluating antibacterial efficacy. Furthermore, *P. pentosaceus* THG-219 enhanced adhesion to host cells while inhibiting pathogen adhesion and demonstrated excellent acid, bile, and heat tolerance. These results suggest that *P. pentosaceus* THG-219 possesses strong stability and adaptability for cosmetic formulations and support its potential as a promising functional ingredient in the cosmetic industry.

## Figures and Tables

**Table 1:** Cultural characteristics of *P. pentosaceus* THG-219 and DSM 20336^T^ and their carbon source utilization.

Acid Produced from	*P. pentosaceus* THG-219	*P. pentosaceus* DSM 20336^T^
Glycerol	w ^2^	− ^3^
D-Xylose	+ ^1^	−
Lactose	−	+
Melibiose	−	+
Sucrose	−	+
D-raffinose	+	−

^1^ Positive, ^2^ Weak positive, ^3^ Negative.

**Table 2:** MIC and MBC of *P. pentosaceus* THG-219 CFS against pathogenic bacteria are depicted. All experiments were performed at least three times, and the data are presented as mean ± standard deviation.

**Strain**	**Minimum Inhibition Concentration (mg/mL)**	
	***S. epidermidis* KCTC 1917**	***S. aureus* KCTC 3881**
*P. pentosaceus* THG-219	1.25	0.625
*P. pentosaceus* KACC 12311	2.5	0.625
	**Minimal Bactericidal Concentration (mg/mL)**	
	***S. epidermidis* KCTC 1917**	***S. aureus* KCTC 3881**
*P. pentosaceus* THG-219	5	5
*P. pentosaceus* KACC 12311	5	5

**Table 3:** Antimicrobial activity as diameter of inhibition zone of THG-219 using disc diffusion assay. Data are presented as mean ± SD of the results from three replicates.

Indicator	Zone of Inhibition (mm)		
	CFS 20 mg/disc	EA 20 mg/disc	AF 20 mg/disc
*S. epidermidis* KCTC 1917	15	20	nd *
*S. aureus* KCTC 3881	19	22	nd *

* nd, non-detected.

## Data Availability

The data presented in this study are available in this paper.
